# Seasonal and geographical distribution of bacillary dysentery (shigellosis) and associated climate risk factors in Kon Tam Province in Vietnam from 1999 to 2013

**DOI:** 10.1186/s40249-017-0325-z

**Published:** 2017-06-21

**Authors:** Hu Suk Lee, T. T. Ha Hoang, Phuc Pham-Duc, Mihye Lee, Delia Grace, Dac Cam Phung, Vu Minh Thuc, Hung Nguyen-Viet

**Affiliations:** 1 International Livestock Research Institute (ILRI), Room 301-302, B1 Building, Van Phuc Diplomatic Compound, 298 Kim Ma Street, Ba Dinh District, Hanoi, Vietnam; 2 Department of Bacteriology, National Institute of Hygiene and Epidemiology, Hanoi, Vietnam; 3 Center for Public Health and Ecosystem Research, Hanoi University of Public Health, Hanoi, Vietnam; 4 Medical Microbiology Department, The Royal Bournemouth Hospital, Bournemouth, UK; 5 ILRI, Nairobi, Kenya; 6 Thanh Do University, Hanoi, Vietnam

**Keywords:** *Shigella*, Bacillary dysentery, Incidence rate, Seasonality, Wet season, Eco-regions, Central regions, Vietnam

## Abstract

**Background:**

Bacillary dysentery (BD) is an acute bacterial infection of the intestine caused by *Shigella* spp., with clinical symptoms ranging from fever to bloody diarrhoea to abdominal cramps to tenesmus. In Vietnam, enteric bacterial pathogens are an important cause of diarrhoea and most cases in children under 5 years of age are due to *Shigella* strains. The serogroups *S. flexneri* and *S. sonnei* are considered to be the most common. The main objective of this study was to, for the first time, assess the seasonal patterns and geographic distribution of BD in Vietnam, and to determine the climate risk factors associated with the incidence of BD in Kon Tum Province, where the highest rate of bacillary dysentery was observed from 1999 to 2013.

**Methods:**

The seasonal patterns and geographic distribution of BD was assessed in Vietnam using a seasonal-trend decomposition procedure based on loess. In addition, negative binomial regression models were used to determine the climate risk factors associated with the incidence of BD in Kon Tum Province, from 1999 to 2013.

**Results:**

Overall, incidence rates of BD have slightly decreased over time (except for an extremely high incidence in 2012 in the north of Vietnam). The central regions (north/south central coast and central highlands) had relatively high incidence rates, whereas the northwest/east and Red River Delta regions had low incidence rates. Overall, seasonal plots showed a high peak in the mid-rainy reason and a second smaller peak in the early or late rainy season. The incidence rates significantly increased between May and October (“wet season”) across the country. In Kon Tum Province, temperature, humidity, and precipitation were found to be positively associated with the incidence of BD.

**Conclusions:**

Our findings provide insights into the seasonal patterns and geographic distribution of BD in Vietnam and its associated climate risk factors in Kon Tum Province. This study may help clinicians and the general public to better understand the timings of outbreaks and therefore equip them with the knowledge to plan better interventions (such as improving water, sanitation, and hygiene conditions) during peak seasons. This can, in turn, prevent or reduce outbreaks and onwards transmission during an outbreak.

**Electronic supplementary material:**

The online version of this article (doi:10.1186/s40249-017-0325-z) contains supplementary material, which is available to authorized users.

## Multilingual abstracts

Please see Additional file 1 for translations of the abstract into five official working languages of the United Nations.

## Background

Bacillary dysentery (BD) is an acute bacterial infection of the intestine caused by *Shigella* spp., with clinical symptoms of fever, bloody diarrhoea, abdominal cramps, and tenesmus [1]. The infection can be transmitted by the faecal-oral route (via contaminated food, water, or fomites), or through direct person-to-person contact [2]. Some people may have no visible symptoms, but can still transmit the organisms to other people. It is estimated that there are at least 80 million cases of bloody diarrhoea and 700,000 associated deaths each year globally, with approximately 99% of cases occurring in developing countries [3, 4].

There are four *Shigella* serogroups, each with multiple serotypes: A (*S. dysenteriae*, 12 serotypes); B (*S. flexneri*, six serotypes); C (*S. boydii*, 18 serotypes); and D (*S. sonnei*, one serotype). *S. flexneri* dominates in developing countries, while *S. sonnei* is commonly reported in developed countries [5–7]. A study focusing on eight Asian countries found that *S. flexneri* (50%) and *S. sonnei* (45%) were predominantly identified among 98 *Shigella* isolates [8]. In Vietnam, a hospital study conducted in Hanoi found that *Shigella* strains were the main cause of diarrhoea in children aged under 5 years in the category of enteric bacterial pathogens (such as *Campylobacter, Escherichia coli*, and *Salmonella* spp.) [9]. In terms of strains, *S. flexneri* and *S. sonnei* are considered to be the most common serogroups in Vietnam [10, 11]. A study conducted in Hanoi showed that the incidence of *S. flexneri* was the highest, followed by *S. dysenteriae* serotype I and *S. sonnei* [12].

Previous studies have demonstrated distinct seasonal patterns of BD around the world. In Chinese studies, a seasonal pattern of BD with a peak in the summer and fall was observed in Huzhou, Jinan, and Shenyang [1, 4, 13–15]. In Sweden, a distinct seasonal pattern was also demonstrated, with the highest rate between July and October among travellers returning from overseas [16]. In another study from Bangladesh, seasonal peaks were observed from September to November in Dhaka [17].

To our current knowledge, few studies have been conducted to evaluate the seasonal patterns of BD in Vietnam [11, 18, 19]. The main objective of this study was to, for the first time, assess the seasonal patterns and geographic distribution of BD in Vietnam, as well as to determine the climate risk factors associated with the incidence of BD in Kon Tum Province, where the highest rate of BD was observed from 1999 to 2013.

## Methods

### Study location and data on BD

Vietnam is a long, narrow country located in Southeast Asia. Its estimated population was 90.7 million in 2014 [20]. Due to the length of the country, the weather is significantly different from one region to another. The annual mean temperature ranges from 22 to 29 °C. Generally, there are two distinct seasons: rainy and hot (from May to October) and dry and cool (from November to April). In southern Vietnam, the mean temperature difference between the two seasons is unremarkable with less than 3 °C, whereas more significant differences are observed in the northern part, where temperatures can vary by up to 12 °C between the two seasons. There are, on average, 100 rainy days per year, and the annual mean rainfall ranges between 1500 mm and 2500 mm [20].

Monthly data on BD for each province in Vietnam from 1999 to 2013 were obtained from the annually published books on infections issued by the Ministry of Health [21]. BD is a notifiable disease (one of the 28 pathogens in Vietnam), which is reported on a monthly basis by the preventive medicine networks. A case of BD is determined as a suspected case that meets the World Health Organization (WHO) case definition: a patient presenting with diarrhoea with visible blood in the stool and fever, anorexia, abdominal cramps, and tenesmus [22]. The number of cases is collected by provincial preventive medicine centres, and then reported to the regional preventive medicine institutes and finally to the National Institute of Hygiene and Epidemiology (NIHE). The Ministry of Health then publishes a book on infections each year. Data from these books were double entered into an Excel spreadsheet. In addition, climate data (monthly average temperature [°C], total precipitation [100 mm], and average relative humidity [%]) were also collected during the study period.

### Data analysis

Descriptive statistics of incidence rates for each five-year period (1999–2003, 2004–2008, and 2009–2013) were determined in order to map the geographic distribution of BD. For this analysis, the estimated population was reversely extrapolated from cases and incidence rates (per 100,000) and then used to estimate the incidence rates for each five-year period. In order to take the administrative changes into account for some provinces, namely Lai Châu, Can Tho, and Dak Lak that were each divided into two provinces in 2004, cases and populations were proportionally calculated based on the following years’ data.

**Fig. 1:**
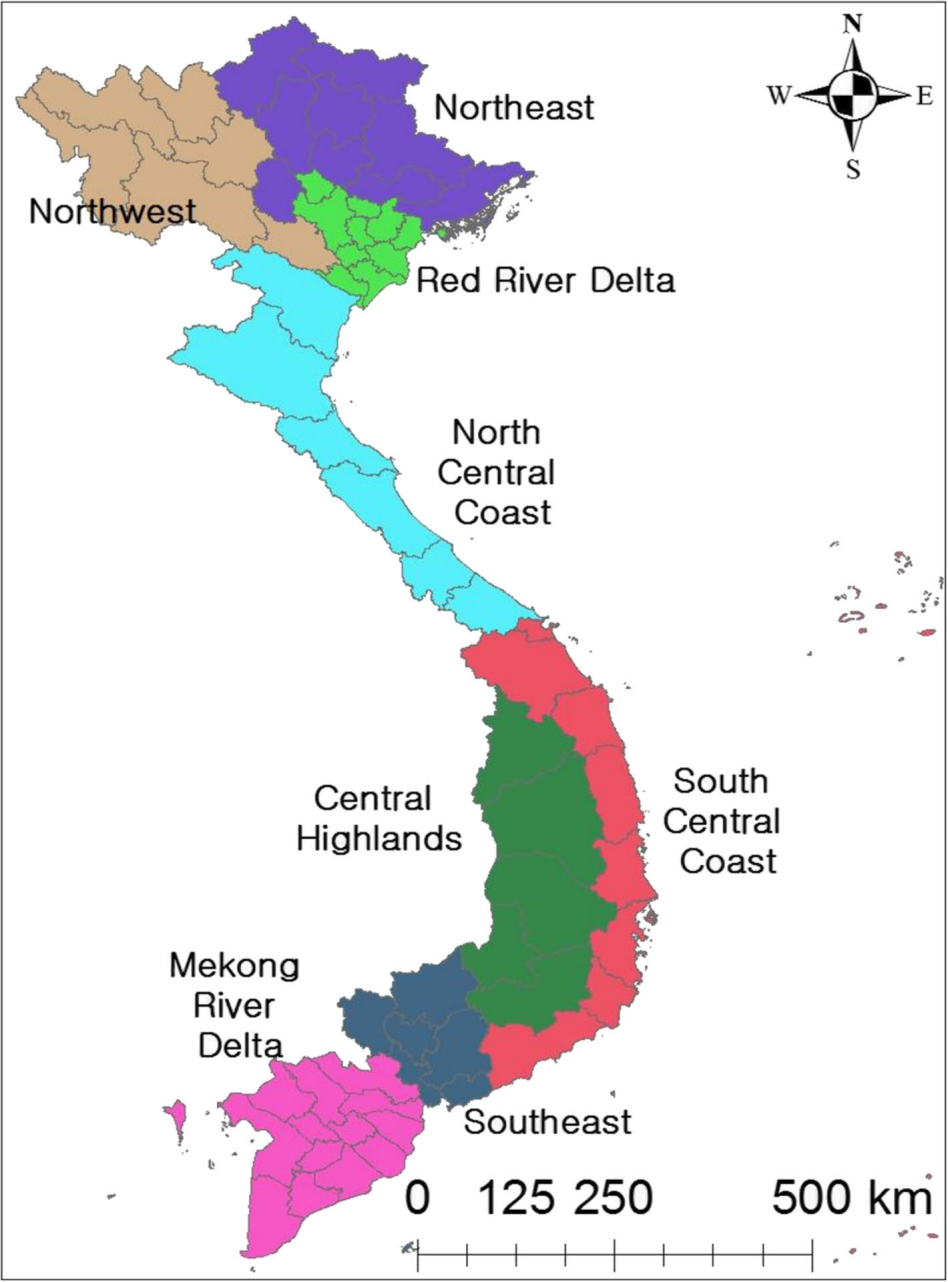
List of regions in Vietnam

For the seasonal pattern analysis, Vietnam was taken to be divided into 58 provinces and five centrally controlled cities existing at the same level as provinces: Hanoi, Ho Chi Minh City, Can Tho, Da Nang, and Hai Phong. These provinces are commonly divided into eight eco-regions based on geographical features and climate conditions (see Fig. 1). Therefore, a, total of 63 provincial level data were classified into eight regions and then the seasonal-trend decomposition procedure based on loess (STL) was conducted for each region to assess the seasonality and trends associated with BD. The STL is a technique to decompose a seasonal time series into trend, seasonal, and remainder on a yearly basis (12 months) [23].

In addition, a seasonal cycle subseries (SCS) plot was used to evaluate the monthly variations, by each region. In order to evaluate the monthly differences, a further statistical analysis (using a univariate negative binomial regression [NBR] model) was conducted [24]. In general, Poisson models are frequently used to analyse count data. However, monthly counts of cases showed evidence of overdispersion (variance greater than the mean), and therefore NBR models that incorporated an overdispersion term (alpha [α]) were preferred to Poisson models [25, 26]. A likelihood ratio test confirmed that α was not zero and that the NBR was more appropriate than the Poisson model (*P* < 0.001). Incidence rate ratios (IRRs) and 95% confidence intervals (*CI*s) were calculated by exponentiation of the regression coefficients. The SCS plot consists of horizontal and vertical lines: a horizontal line for the average incidence rate of each month during the study period and each vertical line from the horizontal line represents the individual incidence rate for that month for each year of the data. For statistical analysis of the average monthly incidence rates, January was used as the reference. If a *P*-value was less than 0.05, it was considered to be significant.

For Kon Tum Province, several multivariable NBR models were explored to evaluate the association between BD and climate variables (temperature, precipitation, and humidity). For the variable selection, the correlations between the three variables (temperature, precipitation, and humidity) were evaluated, while the correlations between the original monthly variables and their one lag (preceding month) were explored in order to assess the potential lagged effect in the model. The linearity of the three variables was investigated using loess smoothing curves. If there was evidence of non-linearity, a quadratic function of the predictor was considered in the model if *P* < 0.05. A random effect for year was included to take into account the unmeasured yearly predictor in the model.

All data were imported into Microsoft Excel 2010 (Microsoft Corporation, Redmond, WA, USA) and analysed using R version 3.2.2 and STATA version 14.0 (StataCorp, College Station, TX, USA). ArcMap in ArcGIS version 10.3 (ESRI, Redlands, CA, USA) was used to create maps.

## Results

### Geographic distribution of BD

**Fig. 2:**
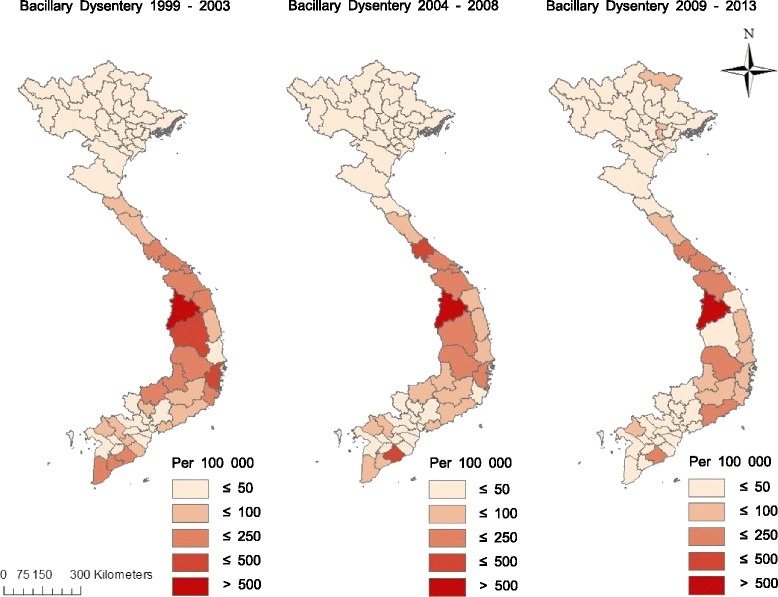
Incidence rates (per 100,000) of BD for each five-year period in Vietnam, from 1999 to 2013

A total of 596,343 (3.98 per 100,000) cases were reported from 1999 to 2013. Overall, incidence rates have slightly decreased over time (see Fig. 2). The central regions (north/south central coast and central highlands) had relatively high incidence rates, whereas the northwest/east and Red River Delta regions has low incidence rates. Within the central regions, the central highlands had the highest incidence rates, ranging between 4.16 and 110.30 per 100,000. Kon Tum Province (population: 0.48 million in 2014) had the highest incidence rates (1203.85 per 100,000 in 1999–2003, 768.69 per 100,000 in 2004–2008, and 674.43 per 100,000 in 2009–2013), followed by Soc Trang and Quang Tri. In contrast, Yen Bai Province (population: 0.78 million in 2014) had the lowest incidence rates (0.94 per 100,000 in 1999–2003, 1.64 per 100,000 in 2004–2008, and 0.68 per 100,000 in 2009–2013) during the study period (see Fig. 2).

### Seasonal patterns of BD incidence

**Fig. 3:**
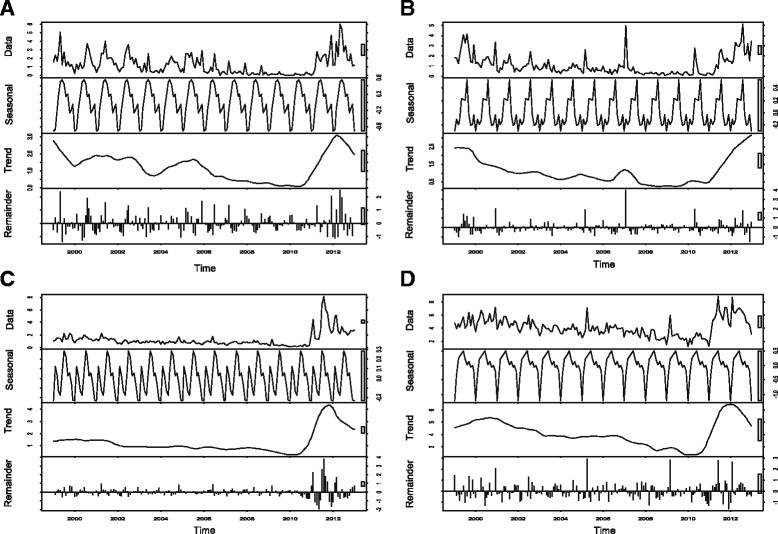
Seasonal-trend decomposition of the monthly incidence rates (per 100,000) of BD, 1999–2013 (each plot has different Y-axis scales). **a** Northwest. **b** Northeast. **c** Red River Delta. **d** North Central Coast

**Fig. 4:**
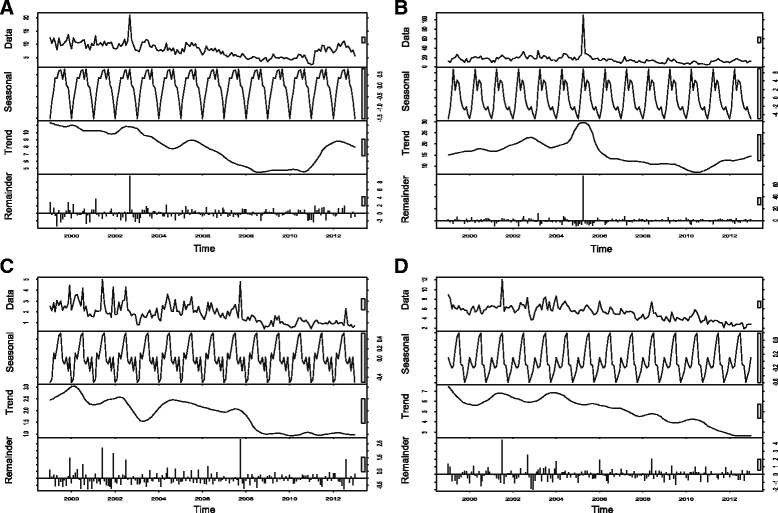
Seasonal-trend decomposition of the monthly incidence rates (per 100,000) of BD, 1999–2013 (each plot has different Y-axis scales). **a** South Central Coast. **b** Central Highlands. **c** Southeast. **d** Mekong River Delta

The incidence rate and trend plots showed the largest fluctuations during the study period; there was a gradual increase after 2011 with a peak in the middle of 2012, and then decreases in five regions (northwest/east, Red Delta, and north/south central coast) (see Figs. 3 and 4: first and third plots). The central highlands – mainly composed of Gia Lai Province, neighbouring province of Kon Tum – had relatively high incidence rates (average: 32.44 per 100,000), with an extreme peak in April 2005.

The STL plots of seasonality indicated a strong seasonal pattern with multiple peaks in each region during the study period (see Figs. 3 and 4: second plots). Overall, seasonal plots showed a higher peak in the mid-rainy reason and a second peak in the early or late rainy season.

**Fig. 5:**
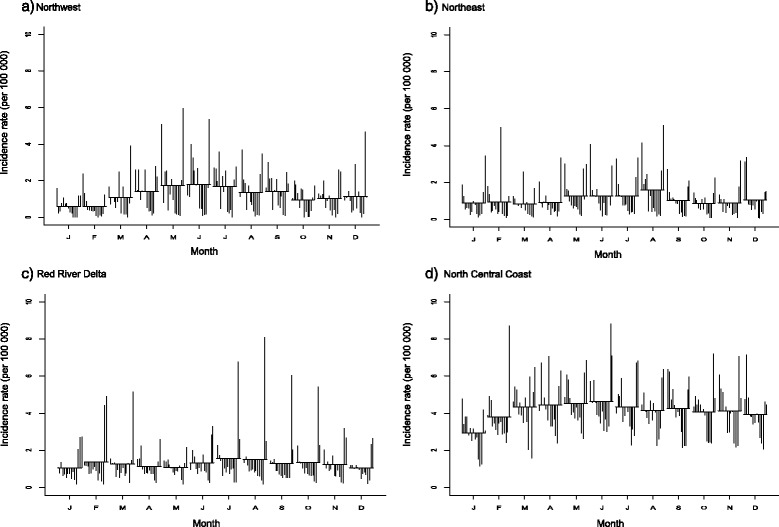
Seasonal cycle subseries plot of the monthly incidence rates (per 100,000) of BD, 1999–2013. **a** Northwest. **b** Northeast. **c** Red River Delta. **d** North Central Coast

**Fig. 6:**
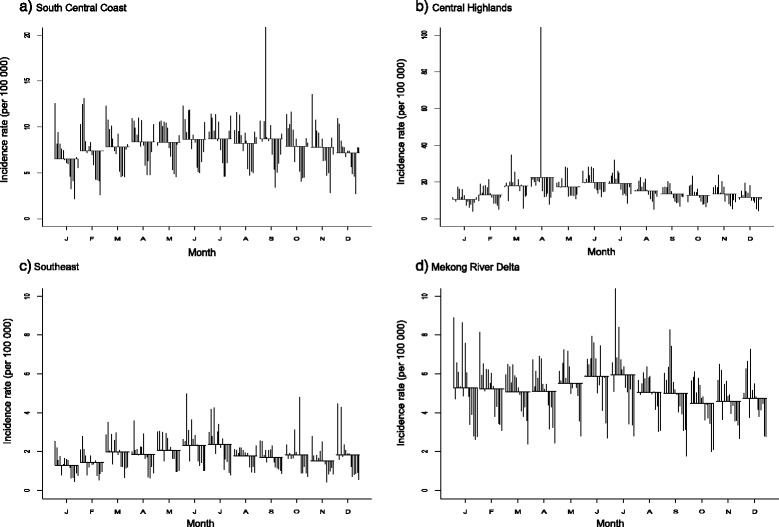
Seasonal cycle subseries plot of the monthly incidence rates (per 100,000) of BD, 1999–2013 (each plot has different Y-axis scales). **a** South Central Coast. **b** Central Highlands. **c** Southeast. **d** Mekong River Delta

The SCS plots showed relatively high incidence rates between May and August compared to other months across the regions (see Figs. 5 and 6). In the Red River Delta region, the SCS plots appeared to have less monthly variations, whereas other regions revealed an inverted U-shape with the highest rates between April and August.

The univariate NRB analysis showed that there was a significantly increased incidence of BD from April to September compared to January in the northwest region (see Table 1). None of the months showed significant differences in the Red River Delta and Mekong River Delta regions, while there was a significant increased incidence of BD in Augustin the northeast region compared to January. In the north central coast region, all months had significant increased incidence rates compared to January. There were significantly increased incidence rates from April to September (except for August) in the south central coast region. In the central highlands, incidence rates of BD significantly increased from March to August. In the southeast region, significantly increased incidence rates were observed from March to July (except for April).

**Table 1:** Univariate negative binomial regression (NBR) models for the BD incidence rates by month with incidence rate ratio (IRR) and 95% confidence interval (*CI*)

Month	Northwest	Northeast	Red River Delta	North Central Coast	South Central Coast	Central Highlands	Southeast	Mekong River Delta
January	Reference: 1	Reference: 1	Reference: 1	Reference: 1	Reference: 1	Reference: 1	Reference: 1	Reference: 1
February	1.14 (0.57–2.27)	1.27 (0.70–2.31)	1.32 (0.81–2.16)	1.29 (1.02–1.62)*	1.11 (0.88–1.41)	1.22 (0.93–1.61)	1.06 (0.76–1.48)	0.95 (0.76–1.21)
March	1.82 (0.92–3.63)	1.16 (0.64–2.11)	1.26 (0.77–2.06)	1.47 (1.17–1.85)*	1.20 (0.95–1.51)	1.66 (1.26–2.17)*	1.45 (1.04–2.02)*	0.93 (0.74–1.18)
April	2.35 (1.18–4.66)*	1.33 (0.73–2.42)	1.11 (0.68–1.82)	1.56 (1.24–1.96)*	1.27 (1.00–1.61)*	2.06 (1.56–2.71)*	1.37 (0.98–1.90)	0.94 (0.74–1.18)
May	2.83 (1.43–5.62)*	1.62 (0.89–2.94)	1.09 (0.67–1.79)	1.59 (1.26–1.99)*	1.28 (1.01–1.62)*	1.66 (1.26–2.19)*	1.52 (1.09–2.12)*	1.02 (0.80–1.28)
June	2.96 (1.49–5.88)*	1.55 (0.85–2.83)	1.29 (0.79–2.11)	1.63 (1.30–2.05)*	1.32 (1.05–1.67)*	1.84 (1.39–2.41)*	1.70 (1.22–2.37)*	1.08 (0.85–1.36)
July	2.90 (1.46–5.77)*	1.56 (0.86–2.84)	1.48 (0.91–2.42)	1.52 (1.20–1.91)*	1.33 (1.06–1.69)*	1.78 (1.36–2.34)*	1.73 (1.24–2.41)*	1.09 (0.86–1.38)
August	2.33 (1.17–4.63)*	1.88 (1.03–3.42)*	1.46 (0.90–2.39)	1.46 (1.16–1.83)*	1.24 (0.98–1.57)	1.42 (1.08–1.86)*	1.32 (0.95–1.84)	0.93 (0.73–1.17)
September	2.34 (1.18–4.64)*	1.27 (0.70–2.31)	1.27 (0.78–2.08)	1.48 (1.17–1.86)*	1.32 (1.04–1.67)*	1.25 (0.95–1.64)	1.25 (0.90–1.74)	0.91 (0.72–1.15)
October	1.56 (0.78–3.10)	1.05 (0.58–1.92)	1.31 (0.80–2.14)	1.41 (1.12–1.78)*	1.21 (0.96–1.54)	1.18 (0.90–1.56)	1.35 (0.97–1.88)	0.82 (0.65–1.04)
November	1.70 (0.85–3.38)	1.11 (0.61–2.02)	1.20 (0.74–1.96)	1.41 (1.12–1.78)*	1.18 (0.93–1.49)	1.24 (0.94–1.64)	1.14 (0.82–1.58)	0.84 (0.67–1.06)
December	1.95 (0.98–3.88)	1.26 (0.69–2.30)	1.04 (0.63–1.70)	1.34 (1.07–1.69)*	1.08 (0.85–1.37)	1.10 (0.84–1.45)	1.33 (0.95–1.84)	0.87 (0.69–1.10)

*statistically significant at *P* < 0.05

### Association between BD incidence and climate factors

In general, the temperature ranged from 6.5 to 36.8 °C, with an average of 24.0 °C. Total monthly precipitation was between 0 mm and 763 mm. Overall, the correlations were relatively high between precipitation and humidity with its lag (see Table 2). Three models were developed, as follows: The first model predicted that a 1 °C increase in temperature in the same month corresponded to a 6% increase in BD incidence, while a 1% increase in humidity corresponded to a 1% increase in BD incidence (see Table 3). The second model showed that 1 °C increase in temperature in the preceding month corresponded to a 7% increase in BD incidence. The third model predicted that a 100 mm increase in precipitation in the same month corresponded to a 4% increase in BD incidence.

**Table 2:** Pearson’s correlation coefficient (r) among climate data with lag 1 in Kon Tum Province from 1999 to 2013 (lag 1: preceding month)

Variable	Monthly average temperature (°C)	Monthly average temperature (°C) (lag 1)	Monthly total precipitation (mm)	Monthly total precipitation (mm) (lag 1)	Monthly average humidity (%)	Monthly average humidity (%) (lag 1)
Monthly average temperature (°C)	1.000					
Monthly average temperature (°C) (lag 1)	0.653	1.000				
Monthly total precipitation (mm)	0.367	0.585	1.000			
Monthly total precipitation (mm) (lag 1)	0.165	0.365	0.565	1.000		
Monthly average humidity (%)	0.171	0.425	0.698	0.647	1.000	
Monthly average humidity (%) (lag 1)	−0.113	0.166	0.425	0.700	0.755	1.000

**Table 3:** Final NBR models of BD incidence rates in Kom Tum from 1999 to 2013

Variable	Adjusted incidence rate ratios (IRRs)	95% *CI*	*P* value
Model 1			
Monthly average temperature (°C)	1.06	1.04–1.09	< 0.001
Average humidity (%)	1.01	1.00–1.01	0.038
Model 2			
Monthly average temperature (°C) – lag1	1.07	1.04–1.10	< 0.001
Model 3			
Monthly total precipitation (100 mm)	1.04	1.01–1.07	0.003

## Discussion

Overall, incidence rates of BD were significantly higher between May and October (“wet season”) across the country, which was consistent with findings from previous studies [11, 18, 19]. The central areas (north/south central coast and central highlands) were at significantly increased risk (highest in July) of BD incidence. During the wet season, the northwest region had a significantly higher risk of BD incidence in June (IRR: 2.96), July (IRR: 2.90), and May (IRR: 2.83) compared to the other regions. This might be attributed to the greatest temperature difference between the two seasons in this area due to high altitudes. The peak period is hotter and wetter, which provides an environment suitable for the growth of bacteria [27, 28].

The central region had the highest rate of BD during the study period. This region is characterized by mountainous areas and tropical monsoons, with clearly distinguishable wet and dry seasons. Poor hygiene and inadequate knowledge on disease prevention likely leads to a high BD disease prevalence in this region. Indeed, the central highlands are where the largest proportions of the minority populations live. In Kon Tum Province, the percentage of minorities is more than 90% compared to 14.3% in Vietnam (the largest ethnic group, Kinh, accounts for 85.7% of the population) [29]. Ethnic minorities, on average, have lower education levels and incomes than the Kinh group; hence their knowledge on hygiene and disease prevention might be limited. In the 1980s and 1990s, minorities from northern provinces migrated to the central region, as free land was provided to immigrants by the Vietnamese government. These minorities are now living in areas where the main water sources are dug wells, shallow wells using a hand pump, rivers, streams, and springs. Surface water is often contaminated as a result of agricultural development and shallow wells may also be contaminated. One study from Dak Lak Province (central highlands) found that water from dug wells showed the highest faecal contamination level when compared to mixed wells and boreholes [30]. Moreover, open defecation or pit latrines may contribute to water sanitation and hygiene issues in this region. In 2015, the use of latrines in the central highlands was only 61% compared to 79% in the Red River Delta and 86% in the southeast, and open defecation is still practiced in this region [31].

This study showed that the incidence rates of BD in the five regions (northwest/east, Red River Delta, and north/south central coast) have increased from 2012. This might be related to the emergence of new serogroups of *Shigella* or an increase of antibiotic resistance [10, 32, 33]. In addition, the introduction of new diagnostic technologies/equipment since 2010 may have led to a higher number of cases being detected.

The association between climate factors and BD incidence in Kon Tum Province, where the highest incidence rates during the study period were recorded, was also evaluated. Temperature, humidity, and precipitation were found to be positively associated with the incidence of BD, which was consistent with previous studies [34–36]. It is possible that high temperatures and humidity provide a good environment for promoting the growth of bacteria [36]. In addition, high precipitation may lead to flooding and affecting water supply systems, and this increases the chances for consumption of contaminated drinking water. However, due to data limitations, we were not able to identify the other potential risk factors for BD (such as level of contamination in water, age, gender, and human behaviour, etc.).

The national surveillance system for BD has been continuously collecting cases at the provincial level in collaboration with well-trained clinicians. The NIHE regularly provides training programs for medical staff at the local preventive medical centres. In addition, biomedical scientists from the clinical microbiology laboratories at the general hospitals (province/region) attend these training programs. Therefore, the clinical judgments among healthcare professionals are quite consistent across the country and reported cases may be representative of all provinces of Vietnam.

A limitation of this study was that BD cases/incidence rates were underestimated due to a lack of medical facilities and public awareness in rural areas. For instance, asymptomatic patients with *Shigella* infection were less likely to be captured in the central surveillance system. Moreover, only 10–20% of cases were confirmed by laboratory. Therefore, it might be possible that patients were misdiagnosed with other enteric bacterial infections (such as amoebic dysentery, or *Campylobacter*, *Salmonella*, and *E. coli* enteric infections) [personal communication]. One study found that the proportions of *Shigella* lab-confirmed cases were highest in children aged 2–10, followed by those aged over 60. In terms of sex, there was not a significant risk difference between male and female cases [11]. We were not able to evaluate the proportions of the circulating serogroups, main source of infection, or the age and gender of the cases due to the limitations of the dataset.

This study indicated that complicated factors affected the incidence of BD, which need further study in order to i) evaluate the association between climate factors and BD, and ii) identify the circulating serogroups and potential risk factors (such as age, contaminated food/water, gender, human behaviour, and socioeconomic conditions) in Vietnam.

## Conclusions

The different geographic regions under study were found to have different seasonal patterns and trends. Overall, Increased incidence rates were observed between May and October, which is the rainy season. Temperature, humidity, and precipitation were found to be associated with the incidence of BD in Kon Tum Province.

This study’s findings provide insights into better understanding the geographical distribution and seasonal patterns of BD in each region, as well as the associated climate risk factors in Kon Tum Province. This study may be useful for clinicians/general public to better understand and gain clues into the timings of outbreaks. In turn, this may lead to an increase in public awareness and the planning of better interventions (such as improved water, sanitation, and hygiene) during peak seasons in order to prevent or reduce further potential outbreaks or onwards transmission during an outbreak.

## Additional file

Additional file 1: Multilingual abstracts in the five official working languages of the United Nations. (PDF 763 kb)

## Abbreviations

BD: Bacillary dysentery
*CI*: Confidence interval
IRR: Incidence rate ratio
NBR: Binomial regression
NIHE: National Institute of Hygiene and Epidemiology
SCS: Seasonal cycle subseries
STL: Seasonal-trend decomposition procedure based on loess
WHO: World Health Organization
