# Supplementary material for: Seasonal and geographical distribution of bacillary dysentery (shigellosis) and associated climate risk factors in Kon Tam Province in Vietnam from 1999 to 2013
Source: Infect Dis Poverty. 2017 Jun 21;6:113. doi: 10.1186/s40249-017-0325-z (PMC5480122; doi:10.1186/s40249-017-0325-z)
Supplement: Additional file 1: — Multilingual abstracts in the five official working languages of the United Nations. (PDF 763 kb) [file 40249_2017_325_MOESM1_ESM.pdf]

Translation of the abstract into the five official working languages of the United Nations

التوزيع الموسمي والجغرافي للزحار العصوي (مرض الشيغيلات) والعوامل المرتبطة بالمخاطر المناخية في مقاطعه كون تام في فيتنام من 1999 إلى 2013

هو سوك لي، ت. ت. ها هوانغ، فوك فأم-دوك، ميهي لي، ديليا غريس، داك كام فونغ، فو مينه ثوك, هونغ نغوين-فيت

الملخص
الخلفية: الزحار العصوي هو عدوي بكتيرية حاده في الأمعاء تسببها نوع من بكتيريا الشيغيلا وترتبط بأعراض سريريه تتراوح من الحمى إلى الإسهال الدموي إلى تشنجات البطن إلى التعنية وفي فيتنام فإن مسببات الامراض البكتيرية المعوية هي سبب هام للإسهال ومعظم الحالات في الأطفال دون سن الخامسة من العمر يكون سببها سلالات بكتيريا الشيغيلا وتعتبر مجموعات الشيغيلا فليكسنيري والشيغيلا سونيي هي الأكثر شيوعا وكان الهدف الرئيسي لهذه الدراسة هو تقييم الأنماط الموسمية والتوزيع الجغرافي للزحار العصوي في فيتنام وذلك وتحديد عوامل المخاطر المناخية المرتبطة بمعدل الإصابة بالزحار العصوي في مقاطعه كون توم وذلك لأول مره حيث لوحظ اعلي معدل للإصابة بالزحار العصوي من 1999 حتي 2013.
الأساليب: تم تقييم الأنماط الموسمية والتوزيع الجغرافي للزحار العصوي في فيتنام باستخدام طريقة اتجاه التحلل الموسمي المبني على الرواسب وبالإضافة إلى ذلك استخدمت نماذج الانحدار ثنائية المتغير السلبية لتحديد عوامل المخاطر المناخية المرتبطة بمعدل الإصابة بالزحار العصوي في مقاطعه كون توم من 1999 إلى 2013.
النتائج: انخفضت معدلات الإصابة بالزحار العصوي انخفاضا طفيفا بمرور الوقت (باستثناء حدوث ارتفاع شديد في 2012 في شمال فيتنام) وكانت معدلات الإصابة في المناطق الوسطي (الساحل الشمالي/الجنوبي والمرتفعات الوسطي) مرتفعة نسبيا في حين انخفضت معدلات الإصابة في المناطق الشمالية الغربية والشرقية ودلتا النهر الأحمر وبوجه عام أظهرت الرسوم البيانية الموسمية ذروة عالية في منتصف موسم الامطار وذروة ثانية أصغر في موسم الامطار المبكر أو المتأخر وزادت معدلات الإصابة بشكل كبير بين مايو و أكتوبر ("موسم الامطار") في جميع انحاء البلد وفي مقاطعه كون توم تبين ان درجه الحرارة والرطوبة وهطول الامطار ترتبط ارتباطا إيجابيا بمعدل الإصابة بالزحار العصوي.
الاستنتاجات: تقدم نتائجنا نظره ثاقبه على الأنماط الموسمية والتوزيع الجغرافي للزحار العصوي في فيتنام وما يرتبط به من عوامل الخطر المناخي في مقاطعه كون توم وقد تساعد الدراسة الحالية الأطباء وعامه الجمهور على فهم توقيت حلات تفشي المرض على نحو أفضل وبالتالي تزويدهم بالمعرفة من أجل التخطيط لتدخلات أفضل (مثل تحسين المياه والمرافق الصحية وظروف النظافة الصحية) وذلك خلال مواسم الذروة وهذا بدوره من الممكن ان يمنع أو يقلل من حالات التفشي وما يليها من الإنتقال اثناء تفشي المرض.

Translated from English version into Arabic by Mohamed Habib

1999-2013 年越南昆嵩省细菌性痢疾的季节和地理分布研究以及相关气候危险因素分析

Hu Suk Lee, T.T. Ha Hoang, Phuc Pham-Duc, Mihye Lee, Delia Grace, Dac Cam Phung, Vu Minh Thuc and Hung Nguyen-Viet

摘要
引言：细菌性痢疾（BD）是一种由志贺氏杆菌引起的急性细菌性肠道感染疾病，临床症状包括发烧、血便、腹痛和里急后重。在越南，肠道细菌性病原体是引起腹泻的一个重要原因，大多数五岁以下的儿童病例都是由志贺氏菌属（*Shigella*）引起的。福氏志贺杆菌（*S. flexneri*）和宋内氏痢疾杆菌（*S. sonnei*）血清型是最常见的。本研究的主要目的是首次评估了越南细菌性痢疾的季节模式和地理分布情况，并确定与昆嵩省细菌性痢疾发病率相关的气候危险因素，该地区在 1999-2013 年间细菌性痢疾的发病率最高。
方法：本研究运用了一种基于黄土的季节性趋势分解方法，对越南细菌性痢疾的季节模式和地理分布进行评估。此外，本研究还运用了负二项式回归模型来确定与 1999-2013 年间昆嵩省细菌性痢疾发病率相关的气候危险因素。
结果：总体而言，随着时间的推移，细菌性痢疾的发病率略有下降（除了 2012 年越南北部的发病率非常高）。中部地区（北部/南部中部海岸和中部高地）的发病率相对较高，而西北/东和红河三角洲地区的发病率低。总体而言，季节性分布图显示，在雨季中期出现了发病高峰，而在雨季的早期或晚期出现了第二个较小的高峰。全国 5 月到 10 月（“雨季”）的发病率显着增加。在昆嵩省，温度、湿度、降水均与细菌性痢疾的发病率呈正相关。
结论：本研究结果为深入了解越南细菌性痢疾的季节模式和地理分布及其在昆嵩省的相关气候危险因素提供了资料。这项研究有助于临床医生和广大民众更好地了解疾病暴发的时间，从而掌握相关知识，在高峰期更好地做好预防措施（如改善饮水、环境卫生和个人卫生

条件）。这反过来又可以防止或减少暴发的发生以及暴发的进一步传播。

Translated from English version into Chinese by Zhou Guan, edited by Pin Yang

Répartition géographique et saisonnière de la dysenterie bacillaire (shigellose) et des facteurs de risques climatiques associés dans la Province de Kon Tum au Vietnam de 1999 à 2013

Hu Suk Lee, T.T. Ha Hoang, Phuc Pham-Duc, Mihye Lee, Delia Grace, Dac Cam Phung, Vu Minh Thuc and Hung Nguyen-Viet

Résumé

Contexte: La dysenterie bacillaire (DB) est une infection bactérienne aiguë de l'intestin causée par *shigella spp.*, avec des symptômes cliniques allant de la fièvre à une diarrhée sanglante, passant par de crampes abdominales et le ténesme. Au Vietnam, des bactéries entériques pathogènes sont une cause importante de la diarrhée et la plupart des cas chez les enfants de moins de cinq ans sont dus à des souches de *shigella*. Les sérogroupes *S. flexneri et S. sonnei* sont considérés comme les plus courants. L'objectif principal de cette étude était, pour la première fois, d'évaluer les tendances saisonnières et la répartition géographique de la DB au Vietnam, et de déterminer les facteurs de risques climatiques associés à l'incidence dans la Province de Kon Tum, où le taux le plus élevé de cette infetion a été observée de 1999 à 2013.
Méthodes: Les tendances saisonnièrs et la répartition géographique de la DB ont été évaluées au Vietnam au moyen d'une procédure de modèle saisonnier fondé sur la décomposition de loess. En outre, les modèles de régression binomiale négative ont été utilisés pour déterminer les facteurs de risques climatiques associés à l'incidence de la DB dans la Province de KonTum de 1999 à 2013.
Résultats: Dans l'ensemble, les taux d'incidence de la DB ont légèrement diminué au fil du temps (sauf pour une très forte incidence en 2012 dans le Nord du Vietnam). Les régions centrales (côte centrale du Nord/Sud et hautes terres du Centre) ont des taux d'incidence relativement élevés, tandis que le Nord-Ouest/Est et les régions de la Rivière Rouge du Delta avaient un faible taux d'incidence. Dans l'ensemble, les diagrammes saisonniers ont montré un grand pic en pleine saison des pluies et un deuxième petit pic en début ou fin de saison des pluies. Les taux d'incidence ont sensiblement augmenté entre Mai et Octobre (saison des pluies) dans tout le pays. Dans la Province de Kon Tum, la température, l'humidité et les précipitations ont été jugées positivement associées à l'incidence de la DB.
Conclusion: Nos résultats apportent des éclaircissements sur les tendances saisonnières et la répartition géographique de la DB au Vietnam et ses facteurs climatiques associés dans la Province de Kon Tum. Cette étude pourrait aider les cliniciens et le public en général à mieux comprendre le calendrier des épidémies et par conséquent acquérir des connaissances afin de mieux planifier les interventions (telles que l'approvisionnement en eau potable, l'assainissement et les conditions d'hygiène) pendant les périodes de pointe. Ceci peut à son tour, prévenir ou réduire les foyers de maladies et au-delà, la transmission lors d'une épidémie.

Translated from English version into French by Kokouvi Kassegne

Сезонное и географическое распространение бактериальной дизентерии (shigellosis) и соответствующих климатических факторов риска в провинции Kon Tam во Вьетнаме с 1999 по 2013 год

Hu Suk Lee, T.T. Ha Hoang, Phuc Pham-Duc, Mihye Lee, Delia Grace, Dac Cam Phung, Vu Minh Thuc and Hung Nguyen-Viet

Реферат

Введение: Бациллярная дизентерия (BD) - это острая бактериальная инфекция кишечника,

вызываемой *Shigella* spp., с клиническими симптомами, начиная от лихорадки кровавого поноса до спазмы в животе с тенезмами. Во Вьетнаме, кишечные бактериальные патогены являются важной причиной diarrhea и большинство случаев болезни у детей в возрасте до пяти лет вызваны *Shigella* strains. *S. flexneri* и *S. sonnei* считаются наиболее распространенными серогруппами. Основной целью данного исследования было, впервые, оценки сезонных моделей и географического распределения BD во Вьетнаме, и определить климатические факторы риска, связанные с заболеваемостью BD в провинции Kon Tum, где самый высокий уровень бактериальной дизентерии наблюдался с 1999 по 2013 год.
Методы: Сезонные закономерности и географическое распределение BD было проведено во Вьетнаме с помощью сезонного тренда процедуры декомпозиции на основе loess. Кроме того, негативные модели биномиальной регрессии были использованы для определения климатических факторов риска, связанных с заболеваемостью BD в провинции Kon Tum, с 1999 по 2013 год.
Результаты: В целом, показатели заболеваемости BD немного сократились со временем (за исключением крайне высокий уровень заболеваемости в 2012 году на севере Вьетнама). Центральные регионы (Северо - /Южно-центральное побережье и Центральное нагорье) имели относительный высокий уровень заболеваемости, в то время как на Северо-западе/Востоке и в дельтах Красной реки имели низкие показатели заболеваемости. В целом, сезонные участки показали высокий пик в середнем дождливом сезоне и второй меньший пик в начале или конце сезона дождей. Заболеваемость значительно увеличивается с мая по октябрь (“влажный сезон”) по всей стране. В провинции Kon Tum, температура, влажность и осадки положительно связаны с заболеваемостью BD.
Заключение: Наши результаты дают представление о сезонных закономерностях и географическом распределении BD во Вьетнаме и соотвественные климатические факторы риска в провинции Kon Tum. Это исследование может помочь врачам и широкой общественности лучше понять тайминги вспышек и, следовательно, вооружить их знаниями лучше планировать вмешательства (такие, как улучшение водоснабжения, санитарии и ситуации гигиены) в пиковые сезоны. Это может, в свою очередь, предотвратить или уменьшить вспышки и передачи далее во время вспышки.

Translated from English version into French by Hao-Qi Zhang

Distribución estacional y geográfica de la disentería bacilar (shigelosis) y los factores de riesgo climáticos asociados en la provincia de Kon Tam en Vietnam de 1999 a 2013

Hu Suk Lee, T.T. Ha Hoang, Phuc Pham-Duc, Mihye Lee, Delia Grace, Dac Cam Phung, Vu Minh Thuc and Hung Nguyen-Viet

Abstracto

Trasfondo: La disentería bacilar (DB) es una infección bacteriana aguda del intestino causada por *Shigella spp*., Con síntomas clínicos que van desde fiebre hasta diarrea sanguinolenta, calambres abdominales y tenesmo. En Vietnam, los patógenos bacterianos entéricos son una causa importante de diarrea y la mayoría de los casos en niños menores de cinco años se deben a cepas de *Shigella*. Los serogrupos *S. flexneri* y *S. sonnei* se consideran los más comunes. El objetivo principal de este estudio fue, por primera vez, evaluar los patrones estacionales y la distribución geográfica de BD en Vietnam y determinar los factores de riesgo climáticos asociados con la incidencia de BD en la provincia de Kon Tum, donde la tasa más alta de disentería bacilar se observó entre 1999 y 2013.
Métodos: Los patrones estacionales y la distribución geográfica de BD se evaluó en Vietnam utilizando un procedimiento de descomposición de tendencia estacional basado en loess. Además, se utilizaron modelos negativos de regresión binomial para determinar los factores de riesgo climático asociados con la incidencia de BD en la provincia de Kon Tum, de 1999 a 2013.
Resultados: En general, las tasas de incidencia de BD han disminuido ligeramente con el tiempo (excepto por una incidencia extremadamente alta en 2012 en el norte de Vietnam). Las regiones centrales (costa central norte/ sur y altiplano central) tuvieron tasas de incidencia relativamente altas, mientras que las regiones del noroeste/ este y del Río Rojo Delta tenían tasas bajas de

incidencia. En general, las gráficas estacionales mostraron un pico alto en la estación media lluviosa y un segundo pico más pequeño en la temporada de lluvias temprana o tardía. Las tasas de incidencia aumentaron significativamente entre mayo y octubre ("estación húmeda") en todo el país. En la provincia de Kon Tum, se encontró que la temperatura, la humedad y la precipitación estaban positivamente asociadas con la incidencia de BD.
Conclusiones: Nuestros hallazgos proporcionan información sobre los patrones estacionales y la distribución geográfica de BD en Vietnam y sus factores de riesgo climáticos asociados en la provincia de Kon Tum. Este estudio puede ayudar a los médicos clínicos y al público en general a comprender mejor los tiempos de los brotes y, por lo tanto, proveerle el conocimiento necesario para planificar mejores intervenciones (como mejorar el agua, la sanidad y las condiciones de higiene) durante las temporadas de máximo crecimiento. Esto puede, a su vez, prevenir o reducir los brotes y la transmisión hacia adelante durante un brote.

Translated from English version into French by Laura C Vicente Rodriguez
